# Cost-effectiveness analysis of adjuvant chemotherapies in patients presenting with gastric cancer after D2 gastrectomy

**DOI:** 10.1186/1471-2407-14-984

**Published:** 2014-12-19

**Authors:** Bin Wu, Te Li, Jian Cai, Yuejuan Xu, Gang Zhao

**Affiliations:** Department of Pharmacy, Ren Ji Hospital, School of Medicine, Shanghai Jiao Tong University, Shanghai, China; Department of Pharmacy, Yuxi People’s Hospital, affiliated with the Kunming Medical College, Nieer Road 21, Yuxi, China; Department of Clinical Oncology, Taixing People’s Hospital, affiliated with the School of Medicine, Yangzhou University, Changzheng Road 1, Taixing, China; Department of Clinical Oncology, the Second Hospital of Nanjing, affiliated with the Medical School of South East University, Zhongfu Road 1, Nanjing, China; Department of General Surgery, Ren Ji Hospital, School of Medicine, Shanghai Jiao Tong University, Shanghai, China

**Keywords:** Gastric cancer, Adjuvant chemotherapy, Economic analysis, Cost-effectiveness

## Abstract

**Background:**

To analyze and compare the economic outcomes of adjuvant chemotherapy with capecitabine plus oxaliplatin (referred to as the XELOX strategy) and of S-1 (the S-1 strategy) for gastric cancer patients after D2 gastrectomy.

**Methods:**

A Markov model was developed to simulate the lifetime disease course associated with stage II or III gastric cancer after D2 gastrectomy. The lifetime quality-adjusted life years (QALYs), associated costs, and incremental cost-effectiveness ratios (ICERs) were estimated. The clinical data were derived from the results of pilot studies. Direct costs were estimated from the perspective of the Chinese healthcare system, and the utility data were measured from end-point observations of Chinese patients. Sensitivity analyses were used to explore the impact of uncertainty on the model’s outcomes.

**Results:**

The combined adjuvant chemotherapy strategy with XELOX yielded the greatest increase in QALYs over the course of the disease (8.1 QALYs compared with 7.8 QALYs for the S-1 strategy and 6.2 for surgery alone). The incremental cost per QALY gained using the XELOX strategy was significantly lower than that for the S-1 strategy ($3,502 vs. $6,837, respectively). The results were sensitive to the costs of oxaliplatin and the hazard ratio of relapse-free survival.

**Conclusion:**

The observations reported herein suggest that adjuvant therapy with capecitabine plus oxaliplatin is a highly cost-effective strategy and more favorable treatment option than the S-1 strategy in patients with stage II or III gastric cancer who have undergone D2 gastrectomy.

## Background

Despite the declining incidence of gastric cancer, it remains the second leading cause of cancer deaths worldwide, with approximately 736,000 deaths and 988,000 new cases each year [1]. East Asia, including China, Korea, and Japan, has one of the highest incidences and mortality rates of gastric cancer [2–4]. D2 gastrectomy is the most widely used surgical treatment for localized gastric cancer, and long-term follow-up has demonstrated a reduction in gastric cancer-related deaths in patients who have undergone D2 gastrectomy compared with D1 gastrectomy [5–7]. As a result, D2 gastrectomy is preferred in Asia for patients presenting with resectable gastric cancer [8].

Although surgery is the most efficient treatment for operable cancer, recurrence may result in cases with poor prognosis. As an important component of resectable gastric cancer therapy, adjuvant chemotherapy could improve patient outcomes, although no consensus about the preferred treatment has been reached [9–11]. According to the guidelines of the National Comprehensive Cancer Network (NCCN), both capecitabine plus oxaliplatin and S-1 are recommended as adjuvant treatments for gastric cancer [12].

Capecitabine is a new oral drug derived from fluorouracil (FU), which is widely used in the therapy of breast, gastrointestinal, and head and neck cancers [13–15]. A regimen consisting of oxaliplatin plus capecitabine is very effective and tolerable in patients with gastric cancer [16]. By contrast, S-1 is an orally active combination of tegafur, gimeracil, and oteracil at a molar ratio of 1:0.4:1 and is used as a novel mode of neoadjuvant chemotherapy [2, 17, 18]. As relatively new adjuvant chemotherapies, both modes of therapy have the potential to decrease recurrence rates and achieve survival benefits for patients compared with surgery alone [19–21]. However, both modes of therapy markedly increase the cost of the entire treatment approach for gastric cancer, and widespread use of these modes would be limited, particularly in health resource-poor countries such as China [22].

Cost-effectiveness analyses can improve resource allocation efficiency by identifying therapies that provide the greatest health benefits at acceptable cost. However, clinical trials that feature health economics assessments are scarce. Therefore, we have used a mathematical modeling approach to conduct health economics analyses.

Within this study, we have developed a health economics model to evaluate the long-term cost-effectiveness of two adjuvant chemotherapies (the adjuvant S-1 and XELOX strategies) compared with surgery alone in patients presenting with gastric cancer and undergoing D2 gastrectomy in China. The model integrates the best available evidence in terms of costs and clinical outcomes resulting from the use of adjuvant therapies to determine whether such strategies truly represent a health budgetary advantage in the context of the Chinese healthcare system.

## Methods

### Analytical overview and model structure

**Figure 1:**
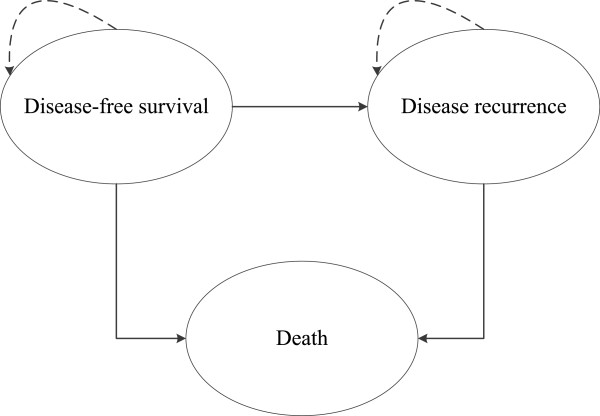
**Schematic depiction of the health economic model.**

In this study, we used a Markov cohort model programmed using R software (version 2.15.1; R Development Core Team, Vienna, Austria) to estimate and compare the lifetime direct medical costs and health benefits associated with surgery only or the use of the adjuvant chemotherapies listed below for patients presenting with gastric cancer after D2 gastrectomy. As shown in Figure 1, there were 3 different health states according to the health outcomes in the model: relapse-free survival (RFS), disease recurrence, and death. The predominant adjuvant therapies for gastric cancer patients, S-1 (the S-1 strategy) and capecitabine plus oxaliplatin (the XELOX strategy), were included in the treatment regimens, and the outcomes of these therapies were compared with surgery only (Surgery strategy). The future costs and benefits were discounted using a 3% annual discount rate.

We assumed that the clinical characteristics of the hypothetical gastric cancer cohorts were consistent with published studies, which had a mean age of 59.5 [2, 23]. The clinical stage of all gastric cancers was II or III, as confirmed by pathology, and all patients underwent curative D2 gastrectomy. The initial health status of the patients was RFS. In the Markov models, one patient was always in one of a series of different health states, called Markov states. All events were represented as movements from one state to another [24]. The cycle length of the model was 1 week. At the completion of each cycle, patients either remained in their assigned health state or progressed to a new health state. The probability of RFS and overall survival (OS) for patients in this model were determined according to the RFS and OS survival data reported in clinical trials [25].

### Clinical data and adjusted indirect comparison

We performed a literature search of the following electronic databases to identify pivotal clinical trials pertaining to adjuvant chemotherapies in patients presenting with gastric cancer and undergoing D2 gastrectomy: PubMed, EMBASE, CINAHL, AltHealthWatch, the Cochrane Library, and the National Library of Science and Technology. The search encompassed the periods from database inception to the end of July 2013. However, no clinical trial directly comparing these three strategies was identified. Therefore, an indirect comparison of key clinical trials was performed. Clinical effectiveness data, including the HR, were extracted from the two pivotal multicenter randomized-controlled clinical trials where patients received surgery only as a common comparator [2, 23]. Each of these trials constituted level 1 evidence [26]. Weibull survival models were fitted to the Kaplan-Meier RFS and OS data for surgery only. The transition parameters and proportions were based on randomized clinical trials to the greatest possible extent (Table 1).

**Table 1:** **Clinical data**

Parameters	Values	Description and reference
**Weibull survival model of RFS for surgery only**		
1-97 weeks	Scale = 0.001554; Shape = 1.202; r^2^ = 0.9955	[2]
Beyond 97 weeks	Scale = 0.02754; Shape = 0.5736; r^2^ = 0.9792	[2]
**Weibull survival model of OS for surgery only**		
1-141 weeks	Scale = 0.00005097; Shape = 1.755; r^2^ = 0.9977	[2]
Beyond 141 weeks	Scale = 0.005259; Shape = 0.8171; r^2^ = 0.9935	[2]
**HR of RFS (compared with surgery only)**		
Adjuvant with S-1	0.653 (95% CI: 0.537-0.793)	[2]
Adjuvant with XELOX strategy^#^	0.58 (95% CI: 0.47–0.72)	[27]
**HR of OS (compared with surgery only)**		[2]
Adjuvant with S-1	0.669 (95% CI: 0.54-0.828)	
Adjuvant with XELOX strategy	0.66 (95% CI: 0.51–0.85)	[27]

^#^We assumed that DFS in the CLASSIC trial was not better than RFS.

The trial reported by the ACTS-GC Group randomized patients presenting with stage II or III gastric cancer who had undergone gastrectomy with extended (D2) lymph-node dissection to surgery followed by adjuvant therapy with S-1 (n = 530) or surgery alone (n = 529). OS at 5 years was 71.7% in the S-1 group and 61.1% in the surgery-alone group (HR, 0.669; 95% CI, 0.540-0.828). RFS at 5 years was 65.4% in the S-1 group and 53.1% in the surgery-alone group (HR, 0.653; 95% CI, 0.537-0.793) [2].

In the CLASSIC trial, 1035 patients were randomized (520 to receive oral capecitabine plus intravenous oxaliplatin and surgery compared with 515 to receive surgery alone). Five-year disease-free survival (DFS) was 68% in the chemotherapy and surgery group and 53% in the surgery-alone group (HR, 0.58, 95% CI 0.47-0.72; P < 0.0001). Five-year OS was 78% in the XELOX group and 69% in the surgery-alone group (HR, 0.66, 95% CI 0.51-0.85; P = .0015) [23, 27].

Indirect comparisons of the three strategies were conducted using the surgery-only survival rate from the reports of the ACTS-GC Group as the reference because the trial reported five-year outcomes. Weibull survival models were fitted to the Kaplan-Meier RFS and OS data from the reports of the ACTS-GC Group for the surgery-only strategy. The estimated Weibull scale (λ) and shape (γ) parameters are shown in Table 1. The Weibull survival curves of the two adjuvant strategies were derived using the HRs, as previously described by Hoyle, M. *et al*. [28]. The RFS and OS HRs between the alternative adjuvant therapy strategies and surgery-only strategy were derived from the previously mentioned published studies.

Patients could die of natural causes during the RFS period at any point beyond the time horizon of the trial follow-ups. The model used a normal life table from the life tables available to WHO member states (2011) to adjust the mortality risk for patients in RFS [29].

### Medical costs and utility

The collection of cost and utility data was approved by the ethics committee of Renji Hospital, and the survey qualified as involving only “minimal risks” to the participants. The survey was completely anonymous, and questionnaire responses were not linked with the participants’ identity in the survey process. Verbal informed consent regarding the goals of the study and willingness to participate was received from the potential respondents. This procedure was approved by the ethics committee of Renji Hospital.

Costs were estimated from the perspective of the Chinese healthcare system and reported in 2013 US dollar equivalents. The following direct medical cost components were considered: treatment-related medicine, monitoring and administration, inpatient care, palliative end-of-life care, and management of serious adverse events. All unit costs of the health resources were estimated using data from the local health system or the National Development and Reform Commission (NDRC) of China [30].

The estimated treatment costs were based on the following schedules for administration of S-1 or XELOX strategies to patients with RFS after D2 gastrectomy. S-1 was administered as a dose of 40 mg/m^2^ twice each day for 4 weeks, followed by 2 weeks of no chemotherapy; this cycle was repeated for 1 year. The treatment regimen for the XELOX strategy consisted of eight 3-week cycles, during which a 2-h intravenous infusion of oxaliplatin was given at a dose of 130 mg/m^2^ on day 1 and oral capecitabine was given at a dose of 1000 mg/m^2^ twice daily on days 1 to 14 of a 3-week cycle. The costs related to SAEs and other costs in the course of adjuvant chemotherapy were derived from a cost analysis study that reported the monetary costs of the two regimens in Chinese gastric cancer patients who had received adjuvant chemotherapy [31]. After cancer progression, salvage chemotherapy and supportive care were available. Approximately 13.5% patients received supportive care, and 86.5% patients received salvage chemotherapy after disease relapse [25]. Based on the literature and expert opinion, the median number of 3-week treatment cycles was seven [25]. The overall costs related to salvage chemotherapy and supportive care were estimated from 133 records of patients who presented with recurrent gastric cancer and received salvage chemotherapy or supportive care at four teaching hospitals in China from January 2010 to April 2013 (the Renji Hospital, the Second Hospital of Nanjing, the Taixing People’s Hospital and the Yuxi People’s Hospital). To estimate the dosage of the therapeutic agents, we assumed that a typical patient weighed 65 kg and had a height of 1.64 m, resulting in a body surface area (BSA) of 1.72 m^2^ [32]. To simplify the model, we assumed that unused drugs in opened vials were discarded. In addition, the current analysis included the cost of palliative end-of-life care. This cost was estimated from 42 records of patients who died from advanced gastric cancer.

The outcome used in this analysis was QALYs. Utility scores ranged from 0.0 to 1.0, with 1.0 representing perfect health and 0.0 representing death. Two panels, one comprising 36 patients with gastric cancer after D2 gastrectomy (disease duration: 4.5 ± 3.7 years) and one comprising 50 patients with recurrent gastric cancer (disease duration: 0.7 ± 0.5 years), were enrolled in the study to complete an interview to empirically measure the utility scores of each health state subgroup in the model. This measurement was performed using the time trade-off (TTO) elicitation technique, in which patients were asked how many additional years they expected to live and how many of those years (if any) they would trade in return for receiving a technology that would guarantee permanent perfect health, based on a standard protocol derived from the methods reported by Redelmeier DA [33]. Health state utility scores are listed in Table 2. QALYs were estimated for each weekly cycle as the number of patients in each health state multiplied by their utility scores. Discounted QALYs that had accrued in each cycle were summed over the lifetime years to determine the total discounted QALYs amassed by the cohort.

**Table 2:** **Base-case cost estimates ($, year 2013 values) and utilities**

Parameter	Median	Range	Description and reference
**Costs**			
Cost of capecitabine per 500 mg	6.6	Fixed	Local charge
Cost of S-1 per 20 mg	9.6	7.9 - 10.1	Local charge
Cost of oxaliplatin per 50 mg	88.9	77.3 - 464.5	Local charge
Cost of follow-up per unit	56.5	42.3 - 70.6	Calculation
Cost of tests per 6-weeks of adjuvant with S-1	197.8	15.9 - 317.5	[31]
Cost of salvage chemotherapy per 3-week cycle	2334.6	1429.3 - 3323.2	Calculation
Cost of palliative end-of-life care	1460.3	1055.3 - 2085.7	Calculation
Cost of supportive care per cycle	115.2	31.7 - 317.5	Calculation
Cost of ADR per 6-week period of adjuvant with S-1	42.3	7.9 - 79.4	[31]
Cost of ADR per 3-week cycle of adjuvant with XELOX strategy	68.9	15.9 - 158.7	[31]
Cost of hospitalization per 3-week cycle of adjuvant with XELOX strategy	373	238.1 - 793.7	[31]
Utilities			
Utility of disease-free survival	0.88	0.8 - 0.97	Measured
Utility of recurrent disease	0.42	0.28 - 0.63	Measured

Key: “&” Values were measured by time trade-off (TTO).

### Sensitivity analysis

To evaluate the uncertainty of parameter values and the robustness of the model, univariate sensitivity analyses were performed for each parameter in the model over the ranges. The results were presented as a tornado diagram based on the impact of the variable on the incremental net health benefit, using 1 × per capita GDP of China as the cost-effectiveness threshold, according to the World Health Organization (WHO) recommendation [34–36]. Probabilistic sensitivity analyses (PSA) were used to simultaneously evaluate the impact of uncertainty across all parameters, in which distributions were assigned to the input parameters of the model (lognormal distributions for costs, beta distributions for probability parameters and utilities). Using these distributions, 1,000 iterations of 1,000 simulated patients were determined. The outcomes projected from all 1,000 simulations were used to plot acceptability curves to estimate the willingness-to-pay (WTP) threshold for an incremental unit of effectiveness.

## Results

### Validation analyses

**Figure 2:**
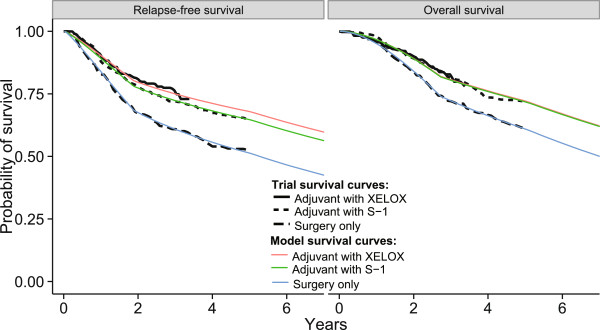
**Calibration curve for RFS and OS.**

The model-derived survival probabilities calculated at specific time points satisfactorily matched those from the clinical trial (Table 3). The RFS and OS data for the different strategies at 3 years and 5 years varied from −5.9% to 2.1% between the model outcomes and the trial data (Table 3). The model outcomes did not exceed the 95% CI of the trial data. Model-derived survival curves did not significantly differ from the results of the clinical trials (Figure 2).

**Table 3:** **Survival probabilities from the model outcomes and trial data**

	Model outcome	Trial data	Difference
Disease-free survival			
Surgery only at 3 years	60.8%	59.6% (95% CI, 54.9 - 64.3%)	1.2%
Adjuvant with S-1 at 3 years	72.3%	72.2% (95% CI, 67.9 - 76.4%)	0.1%
Adjuvant with XELOX strategy at 3 years	75.0%	74% (95% CI, 69 - 79%)	1.0%
Surgery only at 5 years	51.2%	53.1% (95% CI, 48.7 - 57.4%)	−1.9%
Adjuvant with S-1 at 5 years	64.6%	65.4% (95% CI, 61.2 - 69.5%)	−0.8%
Adjuvant with XELOX strategy at 5 years	67.9%	68%	−0.1%
Overall survival			
Surgery only at 3 years	72.2%	70.1% (95% CI, 65.5 - 74.6%)	2.1%
Adjuvant with S-1 at 3 years	80.4%	80.1% (95% CI, 76.1 - 84.0%)	0.3%
Adjuvant with XELOX strategy at 3 years	80.7%	83% (95% CI, 79 - 87%)	−2.3%
Surgery only at 5 years	60.9%	61.1% (95% CI, 56.8 - 65.3%)	−0.2%
Adjuvant with S-1 at 5 years	71.8%	71.7% (95% CI, 67.8 - 75.7%)	0.1%
Adjuvant with XELOX strategy at 5 years	72.1%	78%	−5.9%

### Base-case analyses

**Figure 3:**
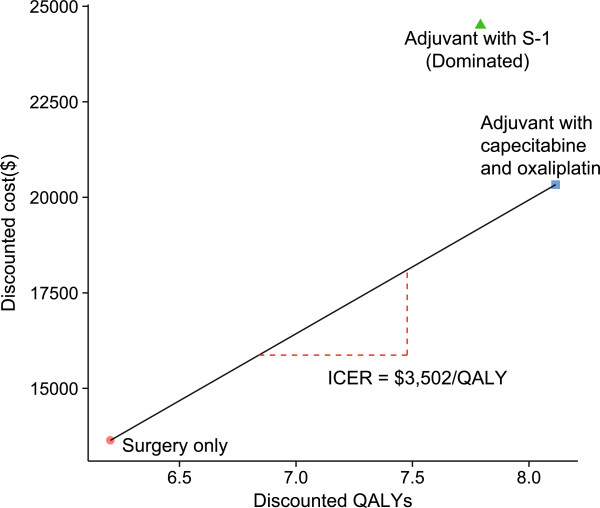
**The cost-effectiveness of strategies for gastric cancer patients.** The oblique line connects surgery only and the most cost-effective strategies. Strategies above the horizontal lines were dominated or extended dominated. In the cost-effective plane, the values of the most incremental cost-effectiveness ratios (ICER) are shown.

Our model estimated the costs and health outcomes of the different strategies. The adjuvant XELOX strategy yielded the greatest increase in QALYs over the course of the disease (8.1 compared to 7.8 QALYs for the S-1 strategy and 6.2 for the surgery-alone strategy), which can be largely explained by the RFS associated with each strategy. Compared to adjuvant therapy with the S-1 strategy, the incremental cost per QALY gained by using the XELOX strategy was significantly lower: $3,502 vs. $6,837 (see Table 4 and Figure 2, and Table 4 and Figure 3, respectively).

**Table 4:** **Summary of cost and outcome results in the base-case analysis**

Strategy	Surgery only	Adjuvant S-1	Adjuvant XELOX
Cost of relapse-free state	445.8	14,776.3	13,468.3
Cost of disease recurrent state	12,248.6	8,984.7	6,166.6
Cost of death from gastric cancer	1,174.8	1,101.4	1,091.7
Total cost ($)	13,638.2	24,503.1	20,331.6
QALYs	8.1	10.8	11.5
Incremental cost per QALY*		6,837	3,502

Key: “*” compared with Surgery only.

### Sensitivity analyses

**Figure 4:**
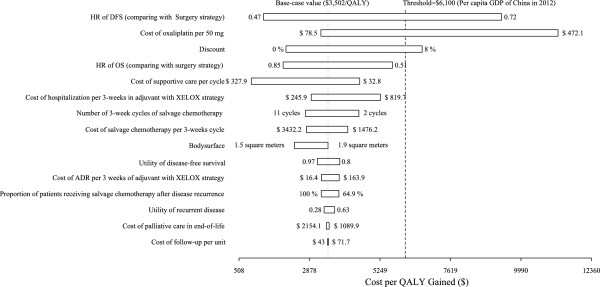
**A tornado diagram representing the net health benefits (in QALYs with WTP = $6,100).** The diagram was determined by a one-way sensitivity analysis of the XELOX strategy vs. surgery only for patients presenting with gastric cancer. The vertical line represents the base-case value for the net health benefit with WTP = $6,100. Key: RFS (relapse-free survival); OS (overall survival); HR (hazard ratio).

The one-way sensitivity analysis revealed that some model variables had a substantial impact on the results of adjuvant therapy using the XELOX strategy compared with surgery alone, including the HR of RFS and the costs of oxaliplatin and supportive care (Figure 4). At the upper boundary of the HR of RFS, the cost of oxaliplatin, the discount rate and the cost per QALY gained ($) exceeds the very cost-effective threshold ($6,100). Other factors, including the HR of OS and costs related to supportive care, salvage chemotherapy and the management of ADRs had moderate or minimal impact on the cost per QALY gained. None of the variable parameters lead to an ICER exceeding a value of $18,300 per additional QALY gained (which represents three times the per capita GDP of China).

**Figure 5:**
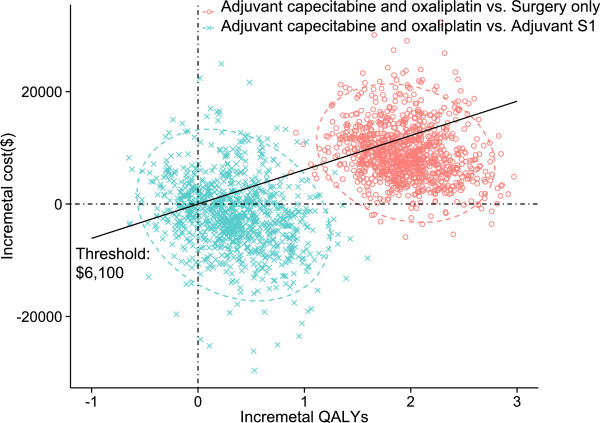
**The probabilistic results of the incremental cost-effectiveness difference.** The XELOX, S-1, and surgery-only strategies were compared. The y-axis represents the incremental costs. The x-axis represents the incremental QALYs gained. The ellipses surround 95% of the estimates. The dots below the ICER threshold (the oblique lines) reflect simulations in which the cost per additional QALY gained for the XELOX strategy was below the ICER threshold.

The plot data from the PSA of 1,000 simulations revealed the probabilities of meeting the ICER thresholds of $6,100 per additional QALY for the XELOX strategy compared with the S-1 and surgery-only strategies (see Figure 5). The probabilities of achieving cost-effectiveness with the XELOX strategy were more than two-thirds compared with the S-1 and surgery-only strategies.

**Figure 6:**
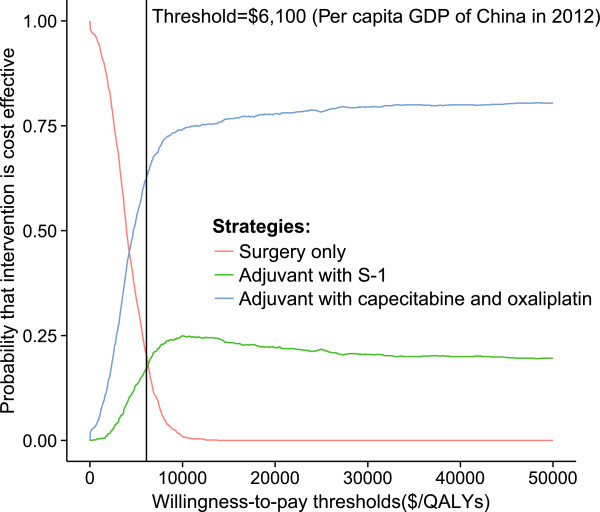
**The cost-effectiveness acceptability curves for the three strategies.** The y-axis indicates the probability that a strategy is cost-effective across the WTP per QALY gained threshold (x-axis). The bold vertical dashed line represents the threshold for China.

The cost-effectiveness acceptability curves (CEACs) revealed the preferred strategies for gastric cancer when accounting for a range of cost-per-QALY thresholds (Figure 6). The CEAC plot demonstrated that the XELOX strategy could achieve nearly two-thirds the likelihood of cost-effectiveness when the threshold level was the per capita GDP of China in 2012 ($6,100).

## Discussion

The current analysis is the first to evaluate the health and economic outcomes of different adjuvant regimens in gastric cancer patients treated with D2 gastrectomy. We determined that adjuvant therapies provide substantial health benefits relative to surgery only by increasing the QALYs (Table 4). This increase in QALYs, in turn, could be contributed to improvements in the RFS and OS rates [2, 23]. The total costs associated with the use of adjuvant chemotherapy for the S-1 and XELOX strategies were $24,503.1 and $18,379.6, respectively, both of which are significantly higher than the cost of surgery only ($13,638.2). However, our cost-effectiveness analysis revealed that the ICERs of the S-1 and XELOX strategies versus the Surgery only strategy are $6,837 and $ 3,502, respectively, per additional QALY gained. According to the WHO recommendation for the cost-effectiveness threshold, both adjuvant strategies are cost-effective because their ICERs are lower than the threshold of $18,300 per additional QALY gained (which represents three times the per capita GDP of China in 2012) [34–36]. In particular, the ICER of the XELOX strategy in the base-case analysis was less than one times the per capita GDP of China in 2012, indicating that adjuvant therapy with the XELOX strategy would be very cost-effective in the Chinese setting based on the WHO recommendation.

As shown in Table 4 and Figure 3, the XELOX strategy may provide greater health benefits and relatively lower costs compared with the S-1 strategy, indicating that the S-1 strategy would be dominant. These results were further confirmed by probabilistic sensitivity analyses and cost-effectiveness acceptability curves (see Figures 5 and 6).

Only three other economic analyses of adjuvant chemotherapy for the treatment of patients with stage II-IIIB gastric cancer with D2 gastrectomy have been conducted, all in Japanese or Chinese settings [37–39]. These reports determined that adjuvant chemotherapy with S-1 or capecitabine plus oxaliplatin after D2 gastrectomy for patients with resectable gastric cancer was a favorable recommendation in accordance with long-term cost-effectiveness compared with D2 gastrectomy alone. On the basis of the current clinical trial and from the perspective of the Chinese healthcare system, the results of these studies are consistent with ours. However, the result reported by Tan C *et al*. suggest that adjuvant treatment with XELOX strategy is a cost-saving strategy over the long term, despite the higher total cost of the XELOX strategy compared with that of surgery alone. Tan C *et al*. determined that the total costs of the surgery-only strategy were $87,004 and $65,894, respectively, far higher than our evaluation ($13,638.2) and the finding ($9,346) reported by Hisashige A *et al*. [37–39]. This discrepancy may be due to the considerable assumption made by Tan C *et al*. that in the situation of tumor recurrence or new occurrences of gastric cancer, cycles of intravenous paclitaxel (at 80 mg/m^2^, three times per week) would be administered every 4 weeks as a first-line chemotherapy for advanced gastric cancer [37, 38]. However, according to the gastric cancer guidelines of the National Comprehensive Cancer Network, clinical trials and our assumptions in previous work, paclitaxel (80 mg/m^2)^ is administered as a second-line chemotherapy for advanced gastric cancer and is repeated weekly for 3 of every 4 weeks [12, 40, 41].

As reported by Tan C *et al*. the cost of oxaliplatin per 50 mg is a substantial consideration [37]. When brand-name oxaliplatin (Eloxatin®, produced by Sanofi-Aventis) was used in the XELOX strategy, the ICER of the XELOX strategy increased to $10,469 per additional QALY gained. The cost of generic oxaliplatin is only one-quarter that of brand-name oxaliplatin, and the generic is now widely accepted and prescribed in Chinese clinical practice. Although the sensitivity analysis indicated that the XELOX strategy was cost-effective (using brand-name oxaliplatin), we suggest the use of generic oxaliplatin to further conserve limited healthcare resources. Another important influential factor was RFS, which would improve the ICER of the XELOX strategy by decreasing the HR. This finding indicates that it is more cost-effective to treat subgroups with more favorable prognostic factors, such as nodal status 1 or 2, with the XELOX strategy compared with the Surgery strategy [23]. The cost-effectiveness threshold of $18,300/QALY was robust and revealed that treatment with an adjuvant therapy using the XELOX strategy was cost-effective.

The results of this analysis must be interpreted carefully within the limitations of the data and study design. First, we used a two-parameter Weibull survival model to extend the tails of survival beyond the follow-up time horizon [42]. Table 3 and Figure 2 show the estimated survival rates fitted to the nonparametric Kaplan-Meier survival rates from the trials, which support the validity of our model. However, there are no long-term (>5 years) RFS and OS data available for patients receiving adjuvant chemotherapy, which could influence the results. Although one-way sensitivity analyses were conducted to evaluate the uncertainty in model outcomes arising from the parameters, this lack of long-term data represents another limitation of our research approach. The current analysis must be updated when long-term outcomes are reported. Second, new therapies are rapidly being developed for managing gastric cancer, including treatment with trastuzumab for HER2-positive gastric cancer; this approach improved the survival of patients with HER2-positive advanced gastric or gastro-esophageal junction cancer [43]. However, these new agents tend to be more expensive than current therapies. Although the current analysis could not trace all medical resources associated with potential new agents in the future, the findings from the one-way sensitivity analyses indicate that the ICER of adjuvant chemotherapy would be improved by increased resource utilization after disease relapse. Third, owing to the absence of head-to-head trials for both of the adjuvant strategies for the adjuvant therapy of gastric cancer patients following D2 gastrectomy compared in this study, an indirect comparison was conducted, another inevitable weakness of the present analysis. The patient characteristics in the CLASSIC trial and in the trial reported by the ACTS-GC Group were assumed to be similar in our indirect comparisons, and the results of the indirect comparison were imputed into the analytical model. Nevertheless, as no data directly comparing the effectiveness of the S-1 and XELOX strategies in large RCTs are available, many investigators worldwide accept indirect comparisons using robust methods. Further studies will be required to directly determine the clinical efficacy of these adjuvant strategies. Fourth, because of the absence of RFS data in the CLASSIC trial, we replaced the RFS data with DFS data to compare the outcomes of the XELOX and S-1 strategies. It could be inferred that the XELOX strategy would have more a favorable RFS than the S-1 strategy because there are more DFS than RFS events; events such as the development of a second primary cancer would lead to more favorable economic outcome [44]. Fifth, the results of this analysis should be carefully interpreted because several factors such as the costs associated with death, the patterns of clinical practice and the availability of health care resources limit the transferability of economic evaluations across jurisdictions. Finally, owing to the nature of the study design, we did not measure exact costs, such as the costs associated with adverse events and palliative care. A cost-of-illness study should be conducted in the future. We believe that our results have theoretical and reference value and provide valuable policy-making data to guide the allocation of health resources in China.

## Conclusions

Taken together, our results indicate that adjuvant therapy with the S-1 or XELOX strategy is a cost-effective option for gastric cancer after D2 gastrectomy and that the XELOX strategy is potentially a very cost-effective alternative to the S-1 strategy or surgery alone, particularly when generic oxaliplatin is used.
